# Short germ insects utilize both the ancestral and derived mode of Polycomb group-mediated epigenetic silencing of Hox genes

**DOI:** 10.1242/bio.201411064

**Published:** 2015-05-06

**Authors:** Yuji Matsuoka, Tetsuya Bando, Takahito Watanabe, Yoshiyasu Ishimaru, Sumihare Noji, Aleksandar Popadić, Taro Mito

**Affiliations:** 1 Department of Life Systems, Institute of Technology and Science, The University of Tokushima Graduate School, 2-1 Minami-Jyosanjima-cho, Tokushima City, Tokushima 770-8506, Japan; 2 Center for Collaboration among Agriculture, Industry and Commerce, The University of Tokushima, 2-24 Shinkura-cho, Tokushima City, Tokushima 770-8501, Japan; 3 Biological Sciences Department, Wayne State University, Detroit, MI 48202, USA; * Present address: Graduate School of Medicine, Dentistry and Pharmaceutical Sciences, Okayama University, 2-5-1 Shikata-cho, Kita-ku, Okayama city, Okayama, 700-8530, Japan.

**Keywords:** Epigenetic silencing, Gene expression, Hox genes, Insect, Polycomb group genes

## Abstract

In insect species that undergo long germ segmentation, such as *Drosophila*, all segments are specified simultaneously at the early blastoderm stage. As embryogenesis progresses, the expression boundaries of Hox genes are established by repression of gap genes, which is subsequently replaced by Polycomb group (PcG) silencing. At present, however, it is not known whether patterning occurs this way in a more ancestral (short germ) mode of embryogenesis, where segments are added gradually during posterior elongation. In this study, two members of the PcG family, *Enhancer of zeste* (*E(z)*) and *Suppressor of zeste 12* (*Su(z)12*), were analyzed in the short germ cricket, *Gryllus bimaculatus*. Results suggest that although stepwise negative regulation by gap and PcG genes is present in anterior members of the Hox cluster, it does not account for regulation of two posterior Hox genes, *abdominal-A* (*abd-A*) and *Abdominal-B* (*Abd-B*). Instead, *abd-A* and *Abd-B* are predominantly regulated by PcG genes, which is the mode present in vertebrates. These findings suggest that an intriguing transition of the PcG-mediated silencing of Hox genes may have occurred during animal evolution. The ancestral bilaterian state may have resembled the current vertebrate mode of regulation, where PcG-mediated silencing of Hox genes occurs before their expression is initiated and is responsible for the establishment of individual expression domains. Then, during insect evolution, the repression by transcription factors may have been acquired in anterior Hox genes of short germ insects, while PcG silencing was maintained in posterior Hox genes.

## Introduction

Though all insects possess highly conserved adult body plans, there are two different ways developmental patterning can be accomplished. In long germ insects, all segments are specified simultaneously at the early blastoderm stage. In contrast, in short or intermediate germ (hereafter collectively called short germ) insects, only the anterior regions are specified at the blastoderm stage. The remaining posterior segments are gradually formed from the most posterior region, termed the growth zone, during posterior elongation. While the short germ type is believed to be the ancestral mode of segmentation in arthropods (reviewed by Davis and Patel, 2002), the actual molecular mechanisms that regulate it have not been identified.

Despite operational differences, previous studies had revealed that canonical functions of segmentation genes are fundamentally conserved between long germ and short germ segmentation (Mito et al., 2005; Mito et al., 2006; Mito et al., 2007). As embryogenesis progresses, a noticeable difference is observed in the expression of Hox genes. In long germ species, such as *Drosophila*, all Hox genes are expressed simultaneously at the blastoderm stage (Akam, 1987; Castelli-Gair, 1998; Bae et al., 2002). In short germ insects, on the other hand, the middle Hox genes, *Sex combs reduced* (*Scr*), *Antennapedia* (*Antp*), and *Ultrabithorax* (*Ubx*), are expressed in the anterior regions preceding initiation of posterior elongation. This is followed by expression of their posterior counterparts, *abdominal-A* (*abd-A*) and *Abdominal-B* (*Abd-B*), which are associated with the formation of posterior segments (Tear et al., 1990; Kelsh et al., 1993; Shippy et al., 1998; Peterson et al., 1999; Zhang et al., 2005). These observed differences in the temporal activation of posterior Hox genes might reflect different regulatory mechanisms of Hox genes.

In *Drosophila*, gap genes function as repressors and provide positional information, determining the anterior border of the Hox gene expression domain. This repressed state of each Hox gene is maintained by Polycomb group (PcG) genes after decay of gap gene activity (Simon et al., 1992; Struhl and Akam, 1985; Jones and Gelbart, 1990). Previous studies reveal that the functions of gap genes as Hox gene repressors are conserved in short germ insects (reviewed by Jaeger, 2011), while the functions of PcG genes have not yet been analyzed in short germ insect development.

Functionally, PcG genes are identified as *trans* regulators that contribute to maintaining the expression patterns of Hox genes in *Drosophila* (Lewis, 1978). PcG gene products comprise 3 different types of complexes termed Polycomb repressive complex (PRC) 1 and 2 and Pleiohomeothic repressive complex (PhoRC). PhoRC, which binds specifically to Polycomb response elements (PREs), recruits PRC2 to PREs. PRC2 then trimethylates histone H3 on lysine 27 (H3K27) residues. This, in turn, provides a platform for recruiting PRC1. PRC1 catalyzes ubiquitination of lysine 119 on histone H2A, leading to silencing of target genes (Grimaud et al., 2006; Müller and Kassis, 2006; Simon and Kingston, 2009; Simon and Kingston, 2013). *Drosophila* PcG mutants exhibit homeotic phenotypes, in which multiple Hox genes are activated in body regions where they should be silent (Simon et al., 1992; Soto et al., 1995). In those mutants, transformation of all segments to posterior segments occurs as a result of misexpressed *Abd-B* (Jones and Gelbart, 1990; Birve et al., 2001). Misexpression of Hox genes starts after establishment of normal expression domains, indicating that PcG genes are involved in maintenance of Hox gene repression but not in initial repression (Simon et al., 1992; Struhl and Akam, 1985; Jones and Gelbart, 1990). Thus, during *Drosophila* embryogenesis, PcG-mediated silencing maintains Hox expression boundaries after they are set in early embryos by gap gene activity (White and Lehmann, 1986; Harding and Levine, 1988; Irish et al., 1989; Reinitz and Levine, 1990; Qian et al., 1991). Concordantly, trimethylation of histone H3 on lysine 27 (H3K27me3) is first detected after germband formation, 4–7 hours after egg laying (AEL) (Tie et al., 2009) and after the stage of assumed gap gene activity.

The PcG gene function to silence Hox genes is conserved in vertebrates. However, the establishment of Hox gene expression domains is different from that in *Drosophila*. In vertebrates, segments are progressively formed from anterior to posterior, along with posterior embryo growth. Hox genes are activated in time-dependent manner, during and after posterior growth, reflecting their positions in the gene cluster. This temporal and spatial collinear activation of Hox genes is accomplished through progressive demethylation of H3K27 in the gene cluster, which is silenced by PcG gene action prior to Hox gene activation (Soshnikova and Duboule, 2009). This sequential histone-demethylation process is regulated by a gradient of retinoic acid signaling (Lee et al., 2007; Agger et al., 2007). Thus, in the regulatory machinery of vertebrate Hox genes, PcG-mediated silencing occurs before Hox gene expression is initiated, and it is responsible for the establishment of individual expression domains. This patterning mechanism differs substantially from *Drosophila*. Because the process of posterior growth in vertebrates might be homologous to that in short germ insects with canonical Wnt signaling and the transcription factor caudal playing crucial roles (Martin and Kimelman, 2009), the regulatory machinery of the posterior Hox genes in short germ insects could be more similar to vertebrates than to *Drosophila*.

To examine this possibility, two members of the PcG family, the *Enhancer of zeste* (*E(z)*) and *Suppressor of zeste 12* (*Su(z)12*) genes, which are essential for histone methyltransferase activity of the PRC2 complex, were investigated in the short germ cricket *Gryllus bimaculatus*. To elucidate their functions in short germ insect development, RNA interference (RNAi)-based functional analyses of these genes were performed in the cricket. Here we discuss how the data provide novel insight into the key transitions and mechanisms governing regulation of Hox genes during animal evolution.

## Results

### Suppression of *Gryllus* PcG genes causes a homeotic phenotype

**Fig. 1.**
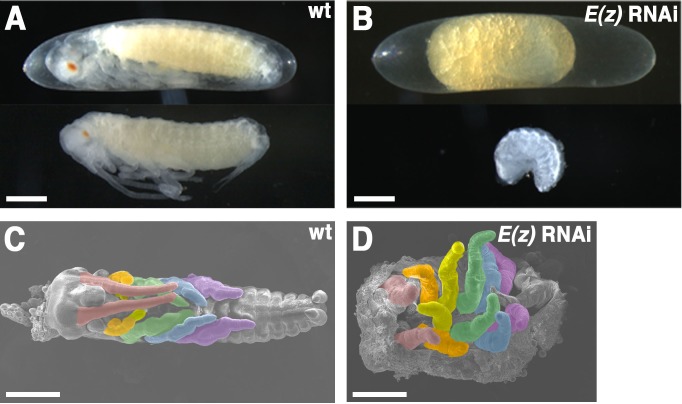
Effects of *E(z)* RNAi during *Gryllus* embryogenesis. (A) Wild type and (B) *E(z)*^RNAi^ embryos ten days after egg laying (AEL). (C) SEM pictures of wild type and (D) *E(z)*^RNAi^ dissected embryos. To enhance visibility, the appendages are artificially colored: antenna (red); maxilla (orange); labium (yellow); T1 leg (green); T2 leg (blue); and T3 leg (purple). Scale bars: 200 µm.

To investigate the functions of PcG genes during embryogenesis, we cloned *E(z)* and *Su(z)12* from *Gryllus* (supplementary material Fig. S1). *E(z)* was first detected by *in situ* hybridization at stage 8 in a ubiquitous pattern (supplementary material Fig. S2A,C). A parental RNAi approach was used to knock down these genes. *E(z)*^RNAi^ embryos exhibited a membrane-enclosed, crescent-shaped body that lacked cuticle formation (Fig. 1B). In these embryos, dorsal closure occurred at the ventral side due to failure in katatrepsis, resulting in an “inside-out” morphology and 100% lethality (Fig. 1B; supplementary material Fig. S3 and Movie 1). As illustrated in Fig. 1D, the phenotype was characterized by a greatly contracted body and by transformation of antennae and mouthparts to leg-like appendages (supplementary material Fig. S5). Two different dsRNAs, *Gb′E(z)_N* and *Gb′E(z)_C*, produced the same morphological phenotypes, excluding the possibility of off-targeting effects by dsRNAs. *Su(z)12*^RNAi^ embryos exhibited a similar but less severe phenotype (supplementary material Fig. S4C). This observation was consistent with real-time quantitative PCR (RT-qPCR) results showing the lesser reduction of *Su(z)12* mRNA levels in *Su(z)12*^RNAi^ embryos compared to *E(z)* mRNA levels in *E(z)*^RNAi^ embryos (supplementary material Fig. S2D,E). For this reason, *E(z)*^RNAi^ embryos were used for further detailed analyses.

The patterns of leg markers, *aristaless* (*al*) (Miyawaki et al., 2002) and *Distal-less* (*Dll*) (Niwa et al., 1997), were determined in *E(z)*^RNAi^ embryos. With the exception of mandibles, the head appendages assumed leg-like expression patterns of *al* compared to wild type (supplementary material Fig. S5A,B). In addition, in the affected head appendages, *Dll* expression patterns resembled those found in thoracic legs with distal and proximal domains (supplementary material Fig. S5C,D). The number of segments was not affected, suggesting that the observed phenotypes were not caused by changes in segment specification mechanisms.

Expression of *wingless* (*wg*), a ventral side marker of legs (Niwa et al., 2000), was restricted to the ventral margin of the elongating limb buds (supplementary material Fig. S5E). However, in *E(z)*^RNAi^ embryos, the *wg* pattern expanded to the dorsal limb margin (supplementary material Fig. S5F). These observations indicated that, in depleted embryos, head appendages (antennae and mouthparts) assume leg-like identities, which also displayed altered dorsoventral polarity.

### *Gryllus E(z)* is involved in histone methylation

**Fig. 2.**
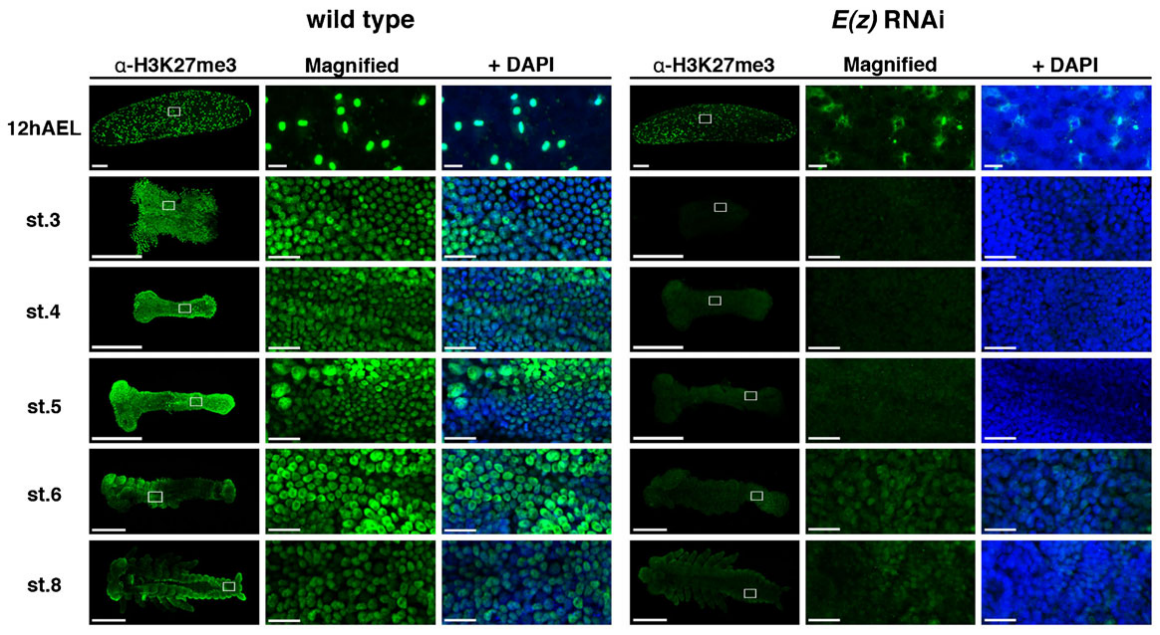
Distribution of H3K27me3 in wild type and *E(z)*^RNAi^ eggs and embryos. Twelve hours AEL, H3K27me3 was detected only in cells on the egg surface. In embryos stage (st.) 3 to st. 8, the H3K27me3 signal was detected throughout the whole embryo. In *E(z)*^RNAi^ eggs and embryos, the H3K27me3 level was reduced, with a slight recovery at stage 6. White boxes indicate regions of higher magnification. Scale bars: 5000 µm in low magnification, and 500 µm in high magnification.

To elucidate whether the observed *E(z)*^RNAi^ phenotype was caused by changes in histone methylation activity, the spatiotemporal distribution of methylated histone 3 on lysine 27 (H3K27me3) was examined. In wild type *Gryllus*, H3K27me3 signals were observed ubiquitously throughout development (Fig. 2, two left-most columns), starting at the early blastoderm stage and continuing through stage 8. In *E(z)*^RNAi^ embryos, H3K27me3 levels were greatly reduced at every stage, although a slight signal was observed at stages 6–8 (Fig. 2, right side), consistent with RT-qPCR results (supplementary material Fig. S2D). These results indicated that the function of *E(z)* in *Gryllus* is to activate and maintain histone methylation, and suggest that the phenotypes observed in Fig. 1B,D may be attributed to changes at the epigenetic level.

### *E(z)*^RNAi^ embryos show anterior misexpression of Hox genes

**Fig. 3.**
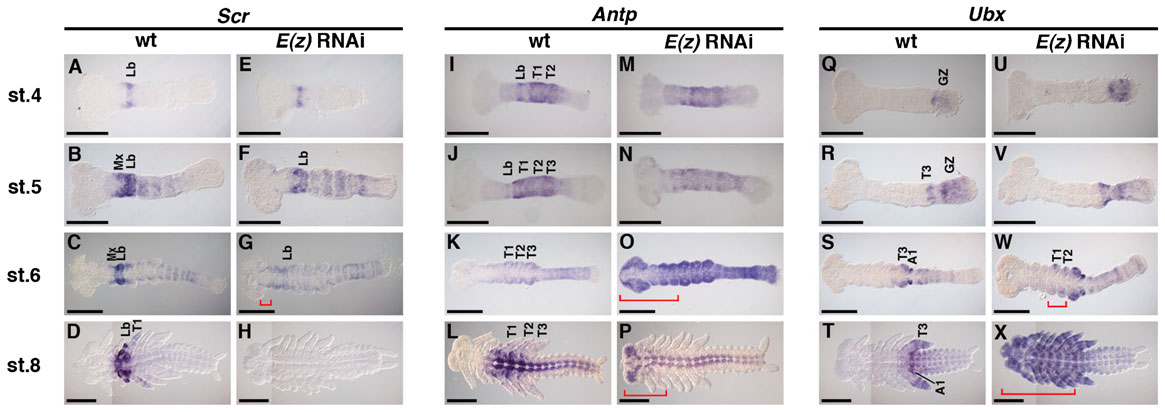
Effects of *E(z)* RNAi on the expression patterns of middle Hox genes. (A–D) Expression patterns of *Scr* in wild type (wt) embryos. Initial expression was restricted to the labrum from stage (st.) 4 to st. 6 (A–C), after which it spread to the T1 segment at st. 8 (D). (E–H) Expression patterns of *Scr* in *E(z)*^RNAi^ embryos. Expression was similar to wt at st. 4 (E), then it became weaker as development progressed until it disappeared completely at st. 8 (F–H). At st. 6, anterior misexpression appeared (red bracket in G). (I–L) Expression patterns of *Antp* in wt. *Antp* was strongly expressed in T1–2 at st. 4 (I), then it expanded into the abdominal segments at st. 5 and st. 6 (J,K). At st. 8, the signal was observed in both the epidermis and central nervous system (CNS) of thoracic and abdominal segments (L). (M–P) Expression patterns of *Antp* in *E(z)*^RNAi^ embryos. (M) Expression was normal at st. 4, then it was reduced at st. 5 (N), followed by ectopic expression encompassing the whole embryo at st. 6. At st. 8, the signal in the epidermis disappeared completely, but it was maintained in the CNS throughout the A–P axis (P). Anterior ectopic expression is shown by the red brackets in O,P. (Q–T) Expression patterns of *Ubx* in wt. (Q) Expression started in the growth zone at st. 4, then it expanded into T1 and abdominal segments at st. 5–8 (R–T). (U–X) Expression of *Ubx* in *E(z)*^RNAi^ embryos. (U,V) The pattern appeared normal until st. 5, after which it expanded into the anterior regions (red brackets in W,X). Abbreviations: Mx: maxilla; Lb: labium; T1–3: thoracic segments 1 to 3; A1: abdominal segment 1; GZ: growth zone. Scale bars: 200 µm.

**Fig. 4.**
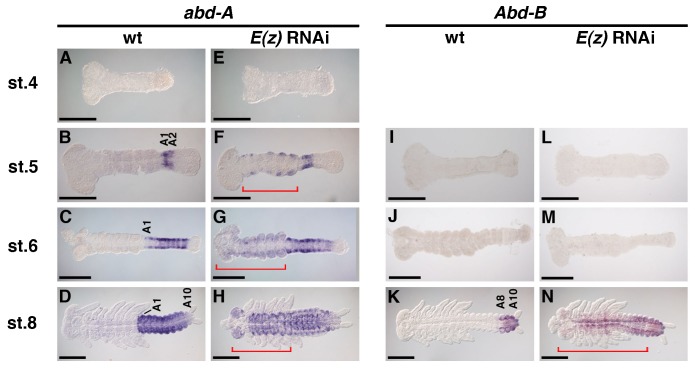
Effects of *E(z)* RNAi on the posterior Hox genes expression patterns. (A–D) Expression patterns of *abd-A* in wild type (wt). Expression started in A1–2 at stage (st.) 5 (B), then it expanded posteriorly to encompass the whole abdomen during st. 6–8 (C,D). (E–H) Expression of *abd-A* in *E(z)*^RNAi^ embryos. *abd-A* expression was not be detected at st. 4 (E). At st. 5 and 6, *abd-A* expanded into anterior regions, as depicted with red brackets (F,G). At st. 8, the signal was observed throughout the ventral regions, the head, and thorax, but was absent from the gnathal appendages and legs (H). (I–K) Expression patterns of *Abd-B* in wt. *Abd-B* was not present until posterior elongation was completed (I,J). Expression started at st. 8 and localized to A8–10 (K). (L–N) Expression of *Abd-B* in *E(z)*^RNAi^ embryos. *Abd-B* expression was absent at st. 5 and 6, similar to wt (L,M). At st. 8, the signal expanded ventrally into the head region and thorax (N). Abbreviations: A1–10, abdominal segments 1 to 10. Scale bars: 200 µm.

To clarify the identity of transformed leg-like structures and determine whether the functions of PcG genes in regulation of Hox genes are conserved in *Gryllus*, the three middle and two posterior Hox genes, *Scr*, *Antp*, *Ubx*, *abd-A* (Zhang et al., 2005), and *Abd-B*, were investigated. Specifically, expression was determined in wild type and *E(z)*^RNAi^ embryos at stage 4 (germband formation stage), stage 5 (just after the initiation of posterior elongation and before A1 and/or A2 are formed), stage 6 (posterior elongation stage and beginning of limb bud formation), and stage 8 (posterior elongation completed). Consistent with the transformation of appendage morphology, all Hox gene patterns were also altered in *E(z)*^RNAi^ embryos (Figs 3 and 4). Similar phenotypes were observed in *Su(z)12*^RNAi^ embryos, although the effects were much milder (supplementary material Fig. S4).

Initial expression domains of the middle genes, *Scr*, *Antp*, and *Ubx*, were established at stage 4 (Fig. 3A,I,Q), and throughout stage 4 and stage 5 expression domains of these three genes were identical in wild-type and *E(z)*^RNAi^ embryos (Fig. 3). Differences in expression were observed at stage 6 (Fig. 3G,O,W). At stage 6, *E(z)*^RNAi^ embryos misexpressed *Scr* in the prospective mandible segment, while *Scr* expression in the prospective labial segment was significantly reduced (Fig. 3G). *Scr* expression was abolished by stage 8 (Fig. 3H). *Antp* expression was reduced in *E(z)*^RNAi^ embryos at stage 5 (Fig. 3N), but it increased in intensity at stage 6 encompassing the whole embryo (Fig. 3O). Neurogenic expression began at stage 8 with anterior ectopic expression (Fig. 3P). Finally, *E(z)*^RNAi^ embryos expressed *Ubx* in the T1 and T2 limb buds at stage 6 (Fig. 3W), which was followed by expansion into the anterior region by stage 8 (Fig. 3X).

Wild-type expression of the two posterior genes, *abd-A* and *Abd-B*, first appeared at stage 5 and stage 8, respectively (Fig. 4B,K). *E(z)*^RNAi^ embryos exhibited anterior expansion of expression, similar to the middle Hox genes (Fig. 3), although ectopic expression of *abd-A* and *Abd-B* appeared almost simultaneously with wild-type expression. For example, in RNAi embryos, the normal (in A1 and A2, in Fig. 4B) and ectopic expression (in the lateral regions of the gnathal and thoracic segments; Fig. 4F) of *abd-A* appeared simultaneously. Ectopic expression continued to expand and encompassed the anterior head segments during stage 6 and stage 8 (Fig. 4G,H). This trend was even more striking in *Abd-B*; ectopic expression reached its full extent at the same stage as wild type expression (Fig. 4N).

In summary, knocking down *E(z)* using RNAi results in anterior misexpression of all examined Hox genes, suggesting that Hox genes were epigenetically silenced through H3K27me3 in the anterior region. In addition, there is a temporal difference in the establishment of ectopic expression domains between the middle and posterior Hox genes. The process is stepwise in the former, but it is simultaneous in the latter.

### PcG genes and gap genes regulate Hox genes independently

*E(z)*^RNAi^ embryos exhibited ectopic expression of *abd-A* in the anterior regions at stage 5 (Fig. 4F). Coincidentally, embryos treated with RNAi directed to the gap gene *hunchback* (*hb*) also exhibit ectopic *abd-A* expression in the prospective gnathal and thoracic segments at stage 5 (Mito et al., 2005). Since PcG genes and *hb* knockdowns altered the regulation of *abd-A* in anterior regions, the genetic relationship between these genes was investigated.

**Fig. 5.**
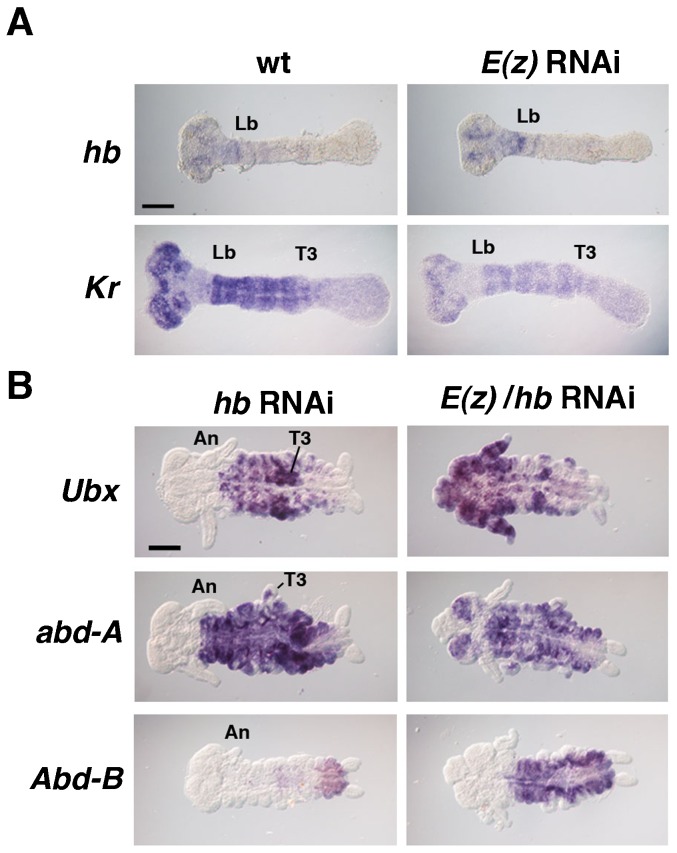
*E(z)* and *hb* regulate posterior Hox genes expression independently. (A) Expression patterns of *hb* and *Kr* were not altered in *E(z)*^RNAi^ embryos at stage (st.) 5. (B) In *hb*^RNAi^ embryos, *Ubx* and *abd-A* were ectopically expressed in the gnathal and thoracic segments, while the *Abd-B* pattern was not altered. In *E(z)*/*hb*^RNAi^ embryos, *Ubx* and *abd-A* were aberrantly expressed in the head regions. The same trend was observed for *Abd-B*, which was ectopically expressed in the gnathal and thoracic segments. Abbreviations: An, antenna; Lb, labium; T3, thoracic segment 3; Scale bars: 100 µm.

The expression patterns of two gap genes, *hb* and *Krüppel* (*Kr*), were investigated in *E(z)*^RNAi^ embryos (Fig. 5A). Results showed that *hb* was not affected. Expression was confined to the prospective mandibular to labial segments (Mito et al., 2005). *Kr* spatial regulation was also preserved, appearing in the labial to T3 segments (Mito et al., 2006). However, overall expression levels were reduced, possibly due to secondary effects of derepression of other genes (Fig. 5A). In summary, spatial expression of *hb* and *Kr* were not altered due to *E(z)* depletion.

Finally, both *E(z)* and *hb* were knocked down simultaneously, and the Hox genes were examined (Fig. 5B). When *hb* was targeted with RNAi, the gnathal and thoracic regions were transformed to abdominal identities due to the expansion of *Ubx* and *abd-A* expression into the gnathal regions (Fig. 5B, left column). Interestingly, *Abd-B* remained unaffected, displaying a wild type pattern. In *hb/E(z)*^RNAi^ embryos, however, ectopic expression of *Ubx* and *abd-A* was observed in the anterior-most head region (antennal and ocular segments) as well. Furthermore, *Abd-B* expression expanded into the gnathal segments (compare Fig. 5B, bottom left and right panels). These observations revealed that the combined *hb/E(z)*^RNAi^ embryos exhibited a combined phenotype that resembled the sum of individual *hb* and *E(z)* knockdowns, indicating that there is no genetic interaction between *hb* and *E(z)*.

### Regional autonomy in the PcG silencing of Hox genes

**Fig. 6.**
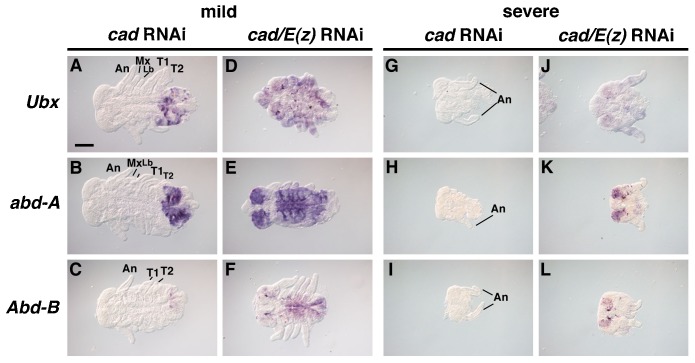
Effects of *E(z)* and *cad* RNAi on the expression pattern of posterior Hox genes. Both mild and severe phenotypes were observed. (A–C) Mild phenotypes of *cad*^RNAi^ embryos. Expression of *Ubx* (A), *abd-A* (B) and *Abd-B* (C) was detected in regions posterior to the T2 segment. (D–F) Mild phenotypes of *cad/E(z)*^RNAi^ embryos. *Ubx* (D), *abd-A* (E), and *Abd-B* (F) were ubiquitously expressed in the whole embryo, consistent with patterns observed in *E(z)*^RNAi^ embryos (Fig. 4). (G–I) Severe phenotypes of *cad*^RNAi^ embryos. (J–L) Severe phenotypes of *cad/E(z)*^RNAi^ embryos. Expression of *Ubx* (J), *abd-A* (K), and *Abd-B* (L) showed anterior expansion. Abbreviations: An, antenna; Mx, maxilla; Lb, labium; T1 and 2, thoracic segments 1 and 2. Scale bar: 100 µm.

To elucidate whether temporal PcG-mediated silencing of Hox genes was related to posterior elongation of the embryo, the *caudal* (*cad*) gene was analyzed. Previous studies have shown that *cad*^RNAi^ embryos lacked posterior segments (Shinmyo et al., 2005), revealing that *cad* is essential for posterior patterning in *Gryllus*. Consistent with previous experiments, the expression of *Ubx*, *abd-A*, and *Abd-B* in *cad*^RNAi^ embryos was determined (Fig. 6). Both mild and severe phenotypes were observed. In mild phenotypes, anterior regions were formed normally, whereas the regions posterior from T2 were missing. In those embryos, *Ubx*, *abd-A*, and *Abd-B* expression was reduced in the remaining posterior regions (Fig. 6A–C). In severe phenotypes, embryos showed only anterior head morphology. Furthermore, the expression of all three genes (*Ubx*, *abd-A*, and *Abd-B*) could not be detected (Fig. 6G–I), suggesting that the growth zone activity was completely abolished in *cad*^RNAi^ embryos.

Treatment with RNAi directed against both *cad* and *E(z)*, produced *cad/E(z)*^RNAi^ embryos, which displayed a phenotype that is a combination of individual *cad* and *E(z)* depleted embryos. In mild phenotypes, *Ubx*, *abd-A*, and *Abd-B* exhibited anterior expansion compared to single *E(z)* RNAi embryos (Fig. 6D–F). Remarkably, this trend became even stronger in the severe phenotype. Indeed, while none of Hox genes were expressed in insects treated with the single *cad* RNAi, they all become overexpressed in the double RNAi mutant (Fig. 6J–L). These observations indicated that PcG-mediated silencing of Hox genes was region-specific and stage-specific, with silencing in the anterior regions occurring independently of posterior region development.

## Discussion

### Functional conservation of the PRC2 complex in *Gryllus*

In the present study, *Gryllus* embryos treated with RNAi directed against *E(z)* displayed reduced H3K27me3 signals during embryogenesis (Fig. 2), suggesting that the E(z) protein plays an important role in histone methylation activity of the PRC2 complex. These observations are consistent with previous work in *Drosophia* (Diptera) and *Bombyx* (Lepidoptera) (Tie et al., 2009; Li et al., 2012). The phenotypes of insects treated with RNAi directed against *Su(z)12*, another component of the PRC2 complex, were similar to *E(z)* RNAi phenotypes, supporting the model of structural and functional conservation of the entire PRC2 complex in *Gryllus* (Orthoptera). Thus, our results strengthen the view that epigenetic regulation machinery involving PcG complexes may be shared among all insect lineages.

There was also a noticeable difference in the developmental dynamics of H3K27me3 in *Gryllus*, which was first detectable at the syncytial blastoderm stage before the germ anlage was formed (12 h AEL; corresponding to stage 4 in *Drosophila*) (Fig. 2). By comparison, H3K27me3 was first detected in *Drosophila* after germband formation at stages 9–11 (Tie et al., 2009). At present, we do not know how *E(z)*-mediated temporal differences in H3K27me3 could affect early embryo development. As indicated by data (supplementary material Fig. S3), one possibility is that H3K27me3 in the early blastoderm may be involved in proper development of extraembryonic tissues and dictate katatrepsis defects observed in *E(z)*^RNAi^ embryos.

### Mode of establishing expression domains varies among *Grylls* Hox genes

As demonstrated in Figs 3 and 4, treatment of *Gryllus* with *E(z)* RNAi resulted in anterior misexpression of *Scr*, *Antp*, *Ubx*, *abd-A*, and *Abd-B*, suggesting that these Hox genes are epigenetically silenced by PcG in the region anterior to each of their individual expression domains. This anterior silencing may be essential for providing proper segment identities in embryos, as the *E(z)*^RNAi^ phenotype exhibited a homeotic transformation of head appendages, with the exception of mandibles, into leg-like structures (Fig. 1). However, while anterior misexpression of those same Hox genes was reported in *E(z)* loss-of-function mutants in *Drosophila*, mutant embryos displayed very different phenotypes; all trunk segments acquired the A8 identity (Jones and Gelbart, 1990). This discrepancy may be due to the fact that ectopic expression of *Abd-B* in *Gryllus* occurs at a germband stage with well-developed leg buds (stage 8), later than the extended-germband stage in *Drosophila* (Simon et al., 1992). This may prevent the inhibition of leg development in *Gryllus*. In addition, ectopic expression of *Scr*, *Antp*, and *Ubx* in early and late appendage buds may activate the leg development program, leading to leg-like transformation of head appendages.

In addition to the different morphological phenotypes of *E(z)* loss-of-function mutants, significant differences in the patterns of Hox gene expression were observed. In *Gryllus E(z)*^RNAi^ embryos, *Scr* expression was lost at stage 8 (Fig. 3H). On the other hand, *Drosophila E(z)* mutant embryos exhibited ectopic expression of *Scr* in the whole embryo at late stages (Soto et al., 1995). It is possible that *Scr* expression in *Gryllus* is repressed by *Ubx*, because *Ubx* is misexpressed in the whole body of late stage *E(z)*^RNAi^ embryos, overlapping potential regions of *Scr* expression. This regulatory relationship may not be conserved in *Drosophila*, suggesting that there may be differences in transcriptional regulation of Hox genes between *Gryllus* and *Drosophila*.

This study revealed another critical difference in PcG silencing of posterior Hox genes (*abd-A* and *Abd-B*) between *Drosophila* and *Gryllus*. In *Drosophila*, the repression by gap genes determines the anterior expression boundary of each Hox gene (in both *Antennapedia* and *Bithorax* complexes). Subsequently, the role of a gap gene is replaced by epigenetic silencing from PcG genes, which, in turn, maintains anterior borders (Simon et al., 1992; Struhl and Akam, 1985; Jones and Gelbart, 1990). Such a stepwise change in the Hox repression system may also apply to regulation of *Scr*, *Antp*, and *Ubx* in *Gryllus*, as indicated by anterior expansion of expression in *E(z)*^RNAi^ embryos (Fig. 3). On the other hand, *Gryllus abd-A* and *Abd-B* were misexpressed at the stage when normal expression appears (Fig. 4), suggesting that PcG silencing is involved in establishing expression domains of these genes. Indeed, *hb* represses *abd-A* expression in anterior (gnathal and thoracic) regions (Mito et al., 2005), as expected if a gap gene is involved in establishing the initial Hox expression domain. However, as shown by embryos treated with RNAi against *hb* and *E(z)*, both genes seemed to act in parallel, not in a stepwise manner (Fig. 5). In addition, *Abd-B* is normally activated after completion of segmentation, when gap gene activity cannot be assumed.

The transcription of *Gryllus* posterior Hox genes seems to be activated during or after posterior embryo elongation and is epigenetically silenced by PcG in the regions outside of their normal expression as a way of establishing their anterior expression boundaries. This mechanism is reminiscent of Hox gene regulation in vertebrates. The initial state of the vertebrate Hox gene cluster is “closed (H3K27me3 positive)” via PcG silencing and subsequently “opened (H3K27me3 negative)” in a temporal manner to induce the expression of a particular Hox gene. Thus, PcG silencing is also required for establishment of the initial expression domains (Soshnikova and Duboule, 2009). This similarity between vertebrates and *Gryllus* with regard to dependence on PcG silencing in establishment of Hox expression domains suggests that this mode may represent the ancestral state in insects.

Activation of *Abd-B* in *Gryllus* may also be regulated in the manner similar to vertebrates. Indeed, this locus may also be silenced by PcG prior to transcription and subsequently turned into an active state when temporal and spatial patterns dictate. It should be noted, however, that inhibition of posterior elongation in *Gryllus* did not affect ectopic expression in the remaining embryonic regions (Fig. 6), implying the existence of autonomous mechanisms for Hox gene silencing and induction of transcriptional activators. Such a transcriptional activator may be upregulated throughout the wild type embryo at a specific developmental stage where Hox genes are expressed. This differs from vertebrates, in which intercellular signals, such as retinoic acid gradients, control where a specific Hox gene is activated or repressed (Kiecker and Lumsden, 2012). Alternatively, the *Gryllus Abd-B* locus might be kept “open” in prospective *Abd-B*-expressing cells throughout posterior embryo elongation until its activation stage, while in more anterior regions the locus might be “closed” prior to *Abd-B* activation. In this case, signals from the posterior growth zone, such as Wnt (Miyawaki et al., 2004), might be involved in inhibiting PcG silencing according to an activity gradient during posterior elongation.

### Evolutionary transition of PcG-mediated silencing of Hox genes

**Fig. 7.**
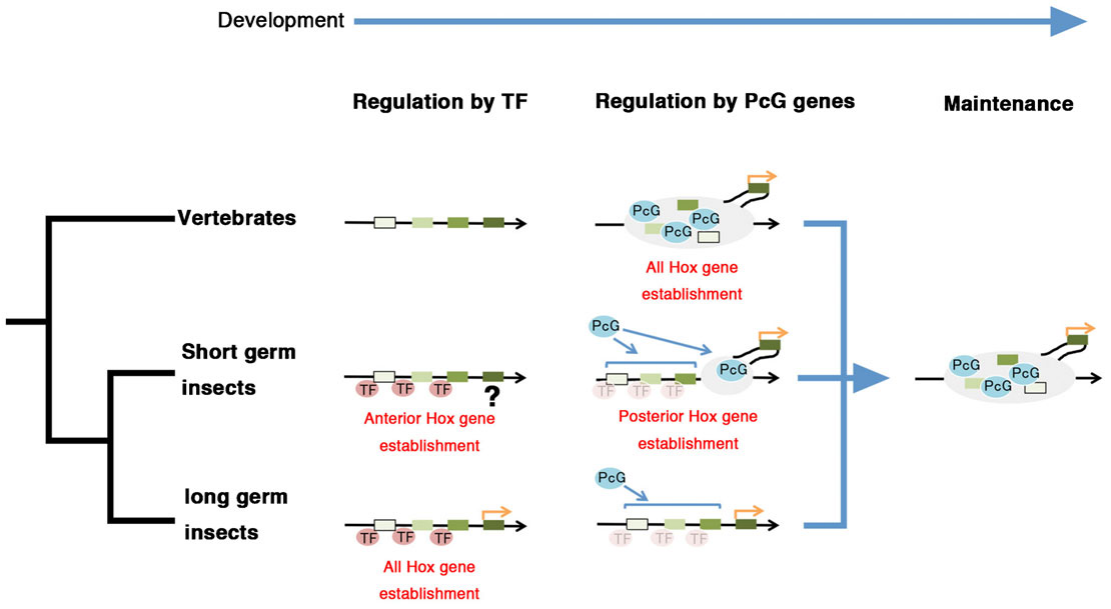
Model of Hox gene regulation machinery during bilaterian evolution. A diagram illustrating the regulation of Hox genes during the development of vertebrates, short germ insects (*Gryllus*), and long germ insects (*Drosophila*). In vertebrates, regulation of all Hox genes is controlled by the PcG complex. During protostome evolution, the introduction of transcription factors (TFs; e.g. gap genes) in short germ insects allowed for the regulation of anterior Hox loci, prior to PcG activity. During the evolution of holometabolous lineages, such as dipterans, all Hox gene loci became regulated by TFs, resulting in apparent stepwise regulation by gap genes and PcG. Different shades of green boxes indicate Hox gene loci. Gray ovals represent PcG repressed chromatin. Orange arrows indicate the start of gene transcription.

Insights from the present and previous studies in vertebrates, *Drosophila*, and *Gryllus*, suggest that an intriguing transition of PcG-mediated silencing of Hox genes occurred during animal evolution (Montavon and Duboule, 2013; Bantignies and Cavalli, 2006). As illustrated in Fig. 7, the ancestral bilaterian state may have resembled the current vertebrate mode, where PcG-mediated silencing of Hox genes occurs before Hox gene expression is initiated, establishing individual expression domains. Then, during insect evolution, repression by transcription factors may have been acquired in anterior Hox genes of short germ insects, while PcG silencing was maintained in posterior Hox genes. During long germ insect evolution, the involvement of transcription factors may have spread to encompass the posterior Hox genes, resulting in the stepwise repression governed by gap and PcG genes that is observed in present day dipterans.

## Materials and Methods

### Animals

*Gryllus bimaculatus* nymphs and adults were reared at 28–30°C with 70% humidity under a 10 light, 14 dark photoperiod, as previously described (Niwa et al., 1997). Fertilized eggs were collected with wet kitchen towels and incubated at 28°C in a plastic dish. Embryos were staged as described previously (Zhang et al., 2005).

### Cloning of the *Gryllus bimaculatus E (z)* and *Su(z)12*

Partial nucleotide sequences of *Gryllus bimaculatus E(z)* and *Su(z)* were identified from cDNA obtained from adult ovaries (Zeng et al., 2013). Partial fragments of *E(z)* and *Su(z)12* were PCR-amplified with gene specific primers and used as double-stranded RNA (dsRNA) templates. The primer sequences for *E(z)_N* and *E(z)_C* (see supplementary material Fig. S1) were 5′-ATACTTGGGCACCAATCCAA-3′ (forward) and 5′-TTCTTCTGGCCTCCCCTTAT-3′ (reverse) and 5′-CTTGGAGTGGACTGCACTGA-3′ (forward) and 5′-CTCGCACAGCAAGATAGCAG-3′ (reverse), respectively. Primer sequences for *Su(z)* are 5′-ATTGAAACGCACCAAGAACC-3′ (forward) and 5′-ATGGGCCACATTCAAGGTAA-3′ (reverse). The cDNA sequences of *Gryllus E(z)* and *Su(z)* were deposited in the DNA Data Bank of Japan (DDBJ) (accession numbers: AB378079; LC005751).

### Parental RNAi

Cloned partial cDNAs of *E(z)* and *Su(z)* were used as templates to prepare dsRNAs. The MEGAScript Kit (Ambion) was used for *in vitro* synthesis of dsRNAs, which were adjusted to a concentration of 20 µM. Parental RNAi treatment was performed by injecting dsRNAs into the body cavity of adult female crickets as described previously (Mito et al., 2005).

### Embryo fixation, whole mount *in situ* hybridization, and immunohistochemistry

Embryo fixation and whole-mount *in situ* hybridization with digoxigenin (DIG)-labeled antisense RNA probes were performed as previously described (Niwa et al., 2000; Zhang et al., 2005). For immunohistochemistry, embryos were fixed with the same methods as *in situ* hybridization (Niwa et al., 2000). Fixed embryos were rehydrated stepwise in 75%, 50%, and 25% solutions of methanol/phosphate-buffered saline + 0.1% Tween (PBT) and PBT for 5 minutes each. Next, embryos were incubated for 1 hour in 1% bovine serum albumin (BSA) (invitrogen)/PBT at room temperature and incubated with a rabbit polyclonal anti-trimethylated H3K27 antibody (Millipore 07-449) diluted 1 to 500 in 1% BSA/PBT overnight at 4°C. After washing with PBT three times, embryos were incubated in 1% BSA/PBT for 1 hour at room temperature and then with Alexa Fluor 488 Goat Anti-mouse IgG(H+L) (Invitrogen) diluted 1 to 500 dilution in 1% BSA/PBT for 1 hour. After washing with PBT three times, embryos were counter stained with DAPI (Sigma) diluted 1 to 1000 in PBT for 10 minutes and then washed with PBT three times. PBT was then substituted by 25% and 50% glycerol/PBT to make embryos transparent for microscopy.

### Real time quantitative PCR

Total RNA was extracted from embryos using ISOGEN (Nippon-Gene). After treatment with DNaseI (Invitrogen), RNA was reverse-transcribed to cDNA using SuperScriptIII reverse transcriptase (Invitrogen). Real-time quantitative PCR was performed using the power SYBR Green PCR Master Kit (Applied Biosystems) and an ABI 7900 Real Time PCR System (Applied Biosystems), as described previously (Nakamura et al., 2008). Primer pairs were: E(z)Fw; 5′-GGCAGATGGCAAAGCAGTGT-3′ and E(z)Rv; 5′-CTTCATGCAGGCAGCATG A-3′; Su(z)12Fw; 5′-ACCGGTGGTGGTGTGTACTT-3′ and Su(z)12Rv; 5′-GCGATAAATCTGCGTTGGTT-3′.

## Supplementary Material

Supplementary Material
