# Supplementary material for: Short germ insects utilize both the ancestral and derived mode of Polycomb group-mediated epigenetic silencing of Hox genes
Source: Biol Open. 2015 May 6;4(6):702–9. doi: 10.1242/bio.201411064 (PMC4467190; doi:10.1242/bio.201411064)
Supplement: Supplementary Material [file supp_4_6_702__index.html]

Supplementary Material 

# Short germ insects utilize both the ancestral and derived mode of Polycomb group-mediated epigenetic silencing of Hox genes

## bio.201411064 Supplementary Material

Yuji Matsuoka et al. doi: 10.1242/bio.201411064

**Files in this Data Supplement:**

- Supplementary Material - Yuji Matsuoka et al. doi: 10.1242/bio.201411064
