# Supplementary material for: Short germ insects utilize both the ancestral and derived mode of Polycomb group-mediated epigenetic silencing of Hox genes
Source: Biol Open. 2015 May 6;4(6):702–9. doi: 10.1242/bio.201411064 (PMC4467190; doi:10.1242/bio.201411064)
Supplement: Supplementary Material [file bio.201411064_bio.201411064-s1.pdf]

Biology Open (2015) 000, 1–8 doi:10.1242/bio.201411064

Supplementary Material

Yuji Matsuoka et al. doi: 10.1242/bio.201411064

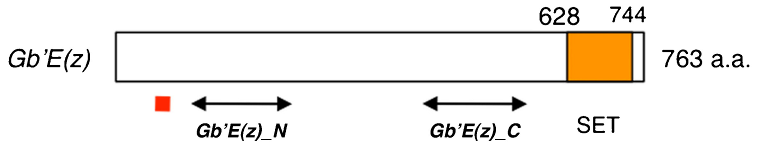


```
Gb’E(z) KHLLMAPSDVAGWGIFLKDSAQKNEFISEYCGEIISQDEADRRGKVYDKYMCSFLFNLNN
Dm’E(z) KHLLMAPSDIAGWGIFLKEGAQKNEFISEYCGEIISQDEADRRGKVYDKYMCSFLFNLNN
        *********:********:.****************************************

Gb’E(z) DFVVDATRKGNKIRFANHSINPNCYAKVMMVNGDHRIGIFAKRAIQPGEELFFDYRY
Dm’E(z) DFVVDATRKGNKIRFANHSINPNCYAKVMMVTGDHRIGIFAKRAIQPGEELFFDYRY
        *******************************.*************************
```

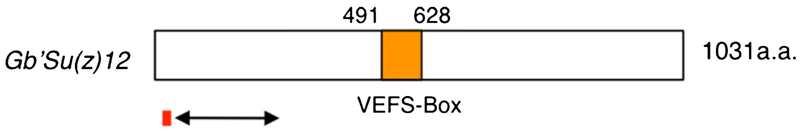


```
Gb’Su(z)12 LELDENEYDGQRPYITGHNRLYHHTTTCLPIYPKEMDIDSEGEHDPKWLQTKTMMMIDDF
Dm’Su(z)12 LELDEDEISNQRSYITGHNRLYHHTETCLPVHPKELDIDSEGESDPLWLRQKTIQMIDEF
           *****:* ..**.************ ****::***:******* ** **: **: ***:*

Gb’Su(z)12 TDVNEGEKELMKMWNLHVMKHGYVGDCQIPLACTMFLESKGKELLMKNLYRNFVLHVCSL
Dm’Su(z)12 SDVNEGEKELMKLWNLHVMRHGFVGDCQLPIACEMFLDAKGTEIVRKNLYRNFILHMCSL
           :***********:******:**:*****:*:** ***::**.*:: *******:**:***

Gb’Su(z)12 FDFGLVSPVTLYNTIQKL
Dm’Su(z)12 FDYGLIAAETVYKTVQKL
           **:**::. *:*:*:***
```

Fig. S1. Domain structure and conservation of *E(z)* and *Su(z)12* in *Gryllus*. Related to Fig. 1. The conserved regions (SET domain and VEFS-Box domain) in E(z) and Su(z)12, are indicated by the orange box. Identical and similarly charged amino acid residues are marked with asterisk and dots, respectively. Arrows show the corresponding regions used to generate dsRNA templates. The red bar shows corresponding regions used for the quantitative RT-PCR.

Biology Open

Biology Open (2015) 000, 1–8 doi:10.1242/bio.201411064

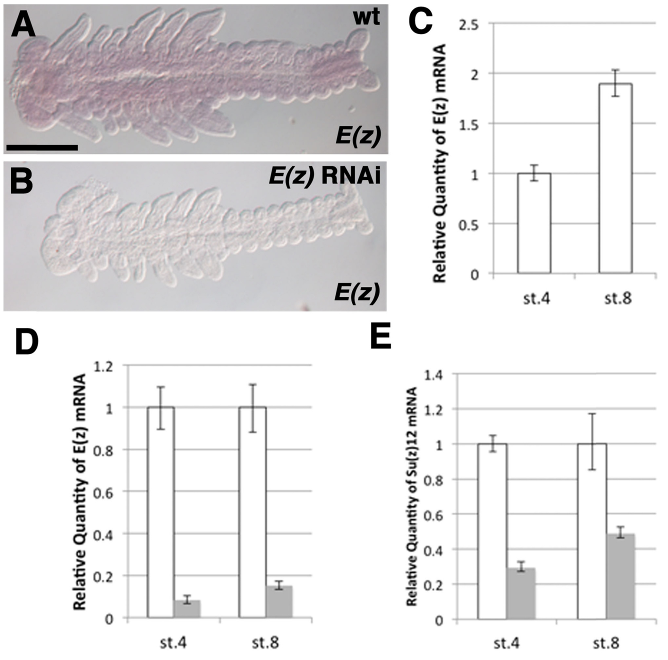

Fig. S2. Expression pattern and level of PcG genes in *Gryllus* embryos. Related to Fig. 1. (A,B) Expression patterns of *E(z)* in wild type and *E(z)*^{RNAi} embryos at stage (st.) 8. (C) Relative amount of *E(z)* mRNA present in st. 4 and st. 8 embryos. At st. 8, E(z) expression levels increased by 89% compared to st. 4. Error bars indicate standard deviation. (D,E) Relative amounts of *E(z)* and *Su(z)12* mRNA in wild type (white bar) and RNAi embryos (gray bar). Scale bar: 200 µm.

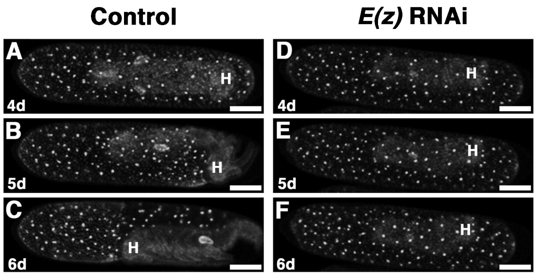

Fig. S3. Effects of *E(z)* RNAi on embryonic movement in transgenic (pBGact-eGFP; Nakamura et al., 2010) embryos. Related to Fig. 1 and supplementary material Movie 1. (A–C) Time-lapse recording of embryonic movement in control embryos undergoing katatrepsis from 4 days after egg laying (AEL) to 6 days AEL. (D–F) In *E(z)*^{RNAi} embryos, katatrepsis did not occur. Scale bars: 200 µm. Abbreviation: H, Head.

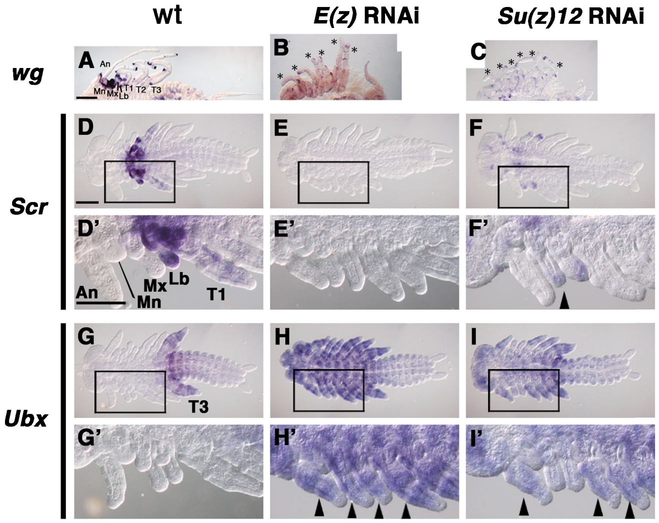

Fig. S4. *Su(z)12*^{RNAi} embryos showed mild misexpression of Hox genes. Related to Figs 1 and 3. (A–C) Morphology of late stage embryos halved at the middle line, along the A–P axis. Asterisks indicate the transformed leg-like appendages. (D–F′) Expression patterns of *Scr* in wild type (wt), *E(z)*^{RNAi} and *Su(z)12*^{RNAi} embryos. In wt, *Scr* was expressed in the labium and T1 segment (D,D′). In *E(z)*^{RNAi} embryos, *Scr* expression was greatly diminished (E,E′). In *Su(z)12*^{RNAi} embryos, *Scr* was expressed in the labium and ectopically expressed in the maxilla (F,F′). (G–I′) Expression patterns of *Ubx* in wt, *E(z)*^{RNAi}, and *Su(z)12*^{RNAi} embryos. In wt, there was no expression of *Ubx* in regions anterior to the T3 segment (G,G′). In *E(z)*^{RNAi} embryos, *Ubx* was ectopically expressed in all appendages (H,H′). In *Su(z)12*^{RNAi} embryos, *Ubx* expression spread to the anterior regions, except in the maxillae and labium (I,I′). Abbreviations: An, antenna; Mn, mandible; Mx, maxilla; Lb, labrum; T1–3, thoracic segments 1–3. Scale bars: 100 µm.

Biology Open (2015) 000, 1–8 doi:10.1242/bio.201411064

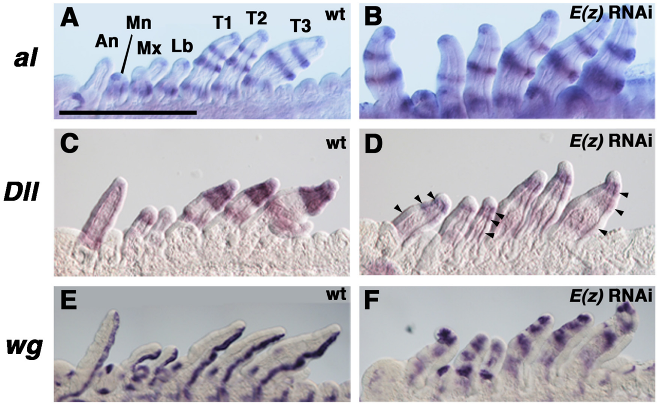

Fig. S5. Effects of *E(z)* RNAi on limb specific genes. Related to Fig. 1. (A,B) Expression pattern of *aristaless* (*al*). In wild type (wt) embryos, *al* was expressed in the most distal regions of developing appendages. In developing legs, *al* was expressed in the femur and tibia (A). In $E(z)^{RNAi}$ embryos, all appendages excluding the mandible showed an *al* expression pattern similar to the limbs of wt embryos (B). (C,D) Expression pattern of *Distal-less* (*Dll*). In wt embryos, *Dll* was expressed in appendages, excluding the mandible (C). The expression pattern was distinct for each appendage. Antenna showed a broad pattern. The maxilla and mandible showed expression in the distal tip. Limbs showed intense expression in distal regions and weak expression in proximal regions. In $E(z)^{RNAi}$ embryos, all appendages, excluding the mandible, showed homogeneous expression patterns of *Dll* with two distal and one proximal domain (shown by arrowheads) (D). (E,F) Expression patterns of *wingless* (*wg*). In wt embryos, *wg* was expressed along the ventral side of each appendage (E). In $E(z)^{RNAi}$ embryos, *wg* was expressed on both the ventral and dorsal sides of each limb (F). Abbreviations: An, antenna; Mn, mandible; Mx, maxilla; Lb, labrum; T1–3, thoracic segments 1–3. Scale bar: 200 µm.

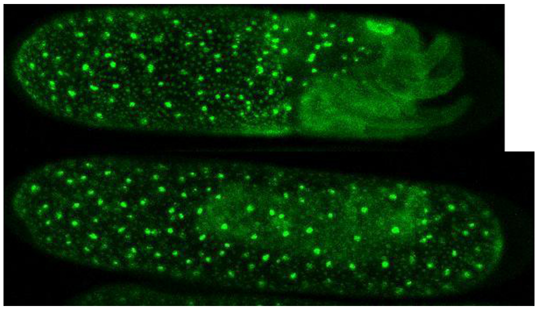

Movie 1. Effects of *E(z)* RNAi on embryonic movement in transgenic (pBGact-eGFP; Nakamura et al., 2010) embryos. Related to Fig. 1. (Upper) Wild type. (Lower) $E(z)^{RNAi}$ embryo.

Biology Open
